# The Causes and Treatment of Diarrhœa—III

**Published:** 1908-11-14

**Authors:** 


### Medicine.

### THE CAUSES AND TREATMENT OF DIARRHŒA—III.

Diarrhœa Due to Local Malignant Disease.

In the next place a few words are necessary in regard to the character of the diarrhœa in cases of venereal or malignant ulceration of the bowel. These two conditions may be taken together, for they may produce very similar symptoms. They are to be distinguished by history and duration and by rectal examination: malignant disease of the bowel generally leaves the lowest inch or more of the intestinal mucosa unaffected, whereas venereal ulceration comes right down to the anus. If, on passing the finger in, normal mucosa is felt for some distance before the ulcer is reached, the lesion is not venereal but malignant. The carcinoma may, of course, occur higher up the colon than the finger can reach; venereal stricture and ulcer, on the other hand, is never so high up that the finger cannot touch it. New growths in the bowel, especially carcinoma of the rectum, are often attended with diarrhœa.

In cancer the diarrhœa is of two types, either of which arising insidiously in a patient of the cancer age must always arouse grave suspicion. The first type is that which has been styled "explosive" diarrhœa, a term which sufficiently explains itself; the other is an apparent rather than a real diarrhœa; twenty times a day the patient may be seized with an intense and urgent desire to defæcate, causing him to fly to the water-closet and pass what he terms a diarrhœic motion, but which really consists of flatus and a little liquid with a very small quantity of fæcal substance in it. The lack of satisfaction experienced by the patient—the feeling that something has been left behind that ought to have come away—is often both a distressing and an extremely suspicious sign of carcinoma, although fæcal accumulations in the rectum may produce a very similar form of diarrhœa, often alternating with constipation. The lesson to be learned from a series of cases is that on no account should rectal examination be omitted in cases of chronic diarrhœa, especially in middle-aged and old people who have not been abroad—that is to say in persons of an age likely to be affected with cancer of the rectum, who have not been exposed to the infection of dysentery.

3. *Nervous Diarrhœa.*—Nervous diarrhœa in its ordinary sense means the condition of affairs in which a patient is so curiously sensitive as regards the bowels that the least excitement or the least anxiety or worry compels a resort to the water-closet for defæcation. The motion itself may be loose, but it is not really diarrhœic. It is simply a question of nervousness of the bowel; mental impressions such as excitement or fear cause such a rapid peristalsis that the colon insists upon emptying itself forthwith. The same kind of thing is more often seen in connection with micturition.

Besides these cases, however, there is the diarrhœa which may result from organic disease of the nervous system with lack of control of the sphincters—to be seen in cases of locomotor ataxia, spastic paraplegia, and the like. Incontinence of the fæces owing to lack of control of the sphincter ani does not necessarily imply diarrhœa, but if there is the least tendency to looseness of the motions, the latter are evacuated far more frequently when there is sphincter paralysis than would be the case in a healthy person.

A third variety of nervous diarrhœa is sometimes to be noted in association with organic disease of some other part of the body than the intestine; in Addison's disease, for example, and in Graves' disease, attacks of apparently causeless diarrhœa are not uncommon. Apparently they are due entirely to nervousness.

Principles of Treatment.

Before a case of diarrhœa can be efficiently treated, it is essential that the cause of the symptom should be diagnosed as exactly as possible.

In the great majority of cases of acute diarrhœa there is an irritant within the bowel; and if it has been decided that this is so, the chief indication is to get rid of this irritant. It is often unnecessary to do anything but wait: the boy who has acute diarrhœa from eating unripe apples will evacuate the latter spontaneously and will be better by next morning. Should active treatment be required, however, in a severe case of the kind it is good to give an ounce of castor oil, to put the patient to bed, to allow no solid food for the rest of the day, to permit barley water or imperial drink to be consumed *ad libitum*, to put warm flannels upon the abdomen if colic is a prominent symptom, to give fifteen minims to half a drachm of tincture of opium by the mouth if the abdominal pains are extreme, and to give a tablespoonful of brandy in three times its bulk of warm water if there is any tendency to collapse.

The most extreme cases of acute diarrhœa that come under observation are those which are associated with vomiting—the summer diarrhœa and vomiting of infants, or the acute vomiting and diarrhœa of ptomaine poisoning. The principles of treatment in these two conditions may be summarised under certain main headings, namely:—

1. To combat the collapse of the patient, and to support by preventing loss of strength.
2. To evacuate from the bowel whatever irritant is present in it.
3. To afford whatever micro-organisms may be present no pabulum to live upon.
4. To inhibit the rate of multiplication of the micro-organisms by the exhibition of antiseptic remedies.
5. To alleviate any irritation there may be of the intestinal mucosa.

(1) The patient must be put to bed at once, and kept there absolutely. A flannel nightgown next the skin will help to prevent chill. The temperature of the air in the room should be at least 68° F.

or 70° F., or even higher, for the bed-clothes will be constantly disturbed by the use of the bedpan, and it is most important that heat should not be abstracted from the patient's body in any way that can be prevented. In the case of small children this is of the very greatest importance; many an infant dies from neglect to prevent loss of body heat during the time when the bed-clothes are partly or wholly withdrawn for purposes of examination, cleansing, and so forth. Too hot a room is far less harmful to a small sick child than is a room that is too cold.

On the question of stimulants opinions still differ. Brandy should not be given indiscriminately, but there is little doubt that many a child's life is saved by it in cases of diarrhœa and vomiting.

The constant loss of fluid in vomits and in motions will rapidly cause dangerous dehydration of the tissues if something is not done to prevent this. The number of vomits and the number of motions may be minimised by the steps described under other headings, and thereby further dehydration may be stopped; but it will be necessary to introduce fresh fluid in many cases. If this can be done by the mouth, all the better; the patient should be allowed to drink barley water or other simple fluid *ad libitum*.

If the stomach is unable to retain even barley water it may be necessary to have recourse to copious injections of fluid viâ the rectum—normal saline, a pint at a time at a temperature of 108° F., will both act as an immediate stimulant by reason of its heat, and also be absorbed in some cases very rapidly. Absorption is more likely to take place if the fluid is given slowly by the continuous siphonage method than if the injection is made with a syringe. Finally, infusion of saline fluid into the cellular tissues of the axillæ may be required, and in many cases of urgency it is strongly indicated. *(To be continued.)*

